# *Lycium barbarum* L.: a potential botanical drug for preventing and treating retinal cell apoptosis

**DOI:** 10.3389/fphar.2025.1571554

**Published:** 2025-03-20

**Authors:** Meng Xiong, Jun Peng, Shunhua Zhou, Qing Gao, Jing Lu, Chen Ou, Houpan Song, Qinghua Peng

**Affiliations:** ^1^ Hunan Provincial Key Laboratory of Traditional Chinese Medicine Diagnostics, Hunan University of Chinese Medicine, Changsha, Hunan, China; ^2^ School of Traditional Chinese Medicine, Hunan University of Chinese Medicine, Changsha, Hunan, China; ^3^ Hunan Provincial Key Laboratory for Prevention and Treatment of Ophthalmology and Otolaryngology Diseases with Chinese Medicine, Hunan University of Chinese Medicine, Changsha, Hunan, China; ^4^ Department of Ophthalmology, The First Affiliated Hospital of Hunan University of Traditional Chinese Medicine, Changsha, Hunan, China; ^5^ School of Medicine, Hunan University of Chinese Medicine, Changsha, Hunan, China

**Keywords:** *Lycium barbarum* L., retinal cells, apoptosis, retinitis pigmentosa, agerelated macular degeneration

## Abstract

Retinal cell apoptosis is the primary pathological process in many retinal diseases, including retinitis pigmentosa and age-related macular degeneration, which can cause severe visual impairment and blindness. *Lycium barbarum L.*, a traditional Chinese medicinal botanical drug, has a long history and extensive application in ophthalmic disease prevention and treatment. This study systematically reviewed the key active metabolites in *L. barbarum L.,* including *L. barbarum* polysaccharides, carotenoids, and flavonoids, that exert retinal protective effects. A comprehensive analysis of the pharmacological effects and underlying molecular mechanisms of *L. barbarum L.* and its active metabolites in the prevention and treatment of retinal cell apoptosis, including essential aspects such as antioxidant activity, anti-inflammatory properties, autophagy regulation, and mitochondrial function preservation, is essential to establish a comprehensive and solid theoretical basis for further investigation of the medicinal value of *L. barbarum L.* in ophthalmology and provide a reference for future research directions.

## Graphical Abstract

**Figure:**
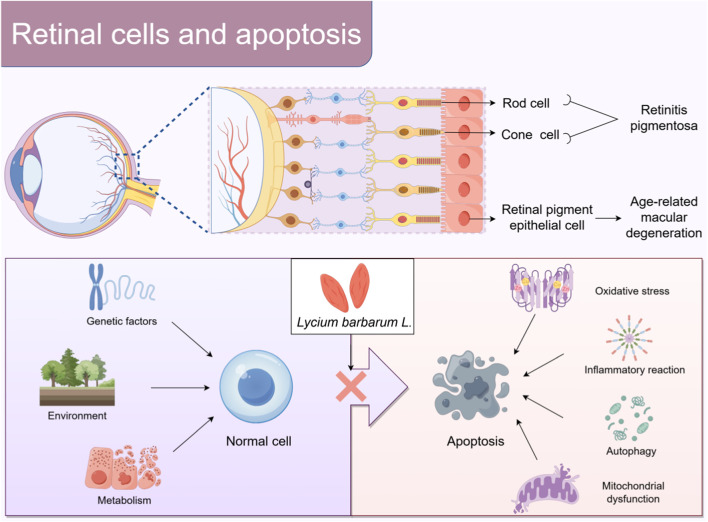
Under the influence of genetic, environmental, and metabolic factors, normal cells may undergo apoptosis, which may be related to oxidative stress, inflammatory response, autophagy dysfunction, and mitochondrial dysfunction. The proposed mechanism suggests that *Lycium barbarum* L. is likely to prevent retinal cell apoptosis By Figdraw.

## 1 Introduction

The photoreceptor cells in the retina, particularly the rod cells and cone cells, are essential components in visual information transmission (Chai et al., 2024). They are responsible for capturing light and converting it into neural signals, which establishes visual perception (Peshenko et al., 2024). Retinal pigment epithelial (RPE) cells function as the “logistical support system” of photoreceptor cells. The RPE absorbs essential nutrients, including glucose, amino acids, and vitamins, from the choroidal capillaries and subsequently transports them to photoreceptor cells to meet their metabolic demands and sustain their normal functions and activities (Li B. et al., 2021). Photoreceptor cells produce substantial metabolic waste, including shed outer disc membrane fragments during the metabolic process. RPE cells phagocytize and degrade these metabolic wastes, maintaining the cleanliness of the retinal microenvironment and preventing metabolic waste accumulation from interfering with retinal function (Zhu et al., 2020). Under physiological conditions, apoptosis influences retinal cell renewal and the clearance of abnormal cells, thereby regulating the maintenance of homeostasis (Yi et al., 2020). Genetic, environmental, metabolic, and other factors can disrupt retinal equilibrium, leading to excessive apoptosis of retinal cells and impairing visual function (Ou et al., 2022). The pathological mechanisms involved in this process are complex, including oxidative stress (Ren et al., 2022), the release of inflammatory mediators (Bishayee et al., 2024), abnormal cellular autophagy (Wang X. et al., 2024), and mitochondrial dysfunction (Wang C. et al., 2024). These mechanisms interact to collectively facilitate retinal cell apoptosis.

Retinal cell apoptosis is the core pathological process in the progression of various retinal degenerative diseases and the main cause of visual impairment and blindness, severely threatening patients’ visual function and quality of life (Chang et al., 2021). Retinitis pigmentosa (RP) (Qiu et al., 2025) and age-related macular degeneration (AMD) (Hu et al., 2024) are representative retinal diseases characterized by retinal cell apoptosis. Their high incidence rate and increasing number of patients have brought a major burden to global health (Fitton et al., 2025; Mosallaei et al., 2025). The strategy for treating retinal cell apoptosis aims to mitigate or prevent this process to protect and restore visual function. While treatment modalities, including antioxidant therapy (Liu Y.chen et al., 2024), gene therapy (Staurenghi et al., 2022), and stem cell transplantation (Maeda et al., 2022), exist, their efficacy and safety require additional validation and optimization (Singh et al., 2020). Therefore, developing new, efficient, and safe prevention and control strategies is imperative.

Goji berries from traditional Chinese medicine (TCM) have attracted much attention for their potential retinal protective effects. The goji berry, *Lycium barbarum* L., is a perennial shrub in the Solanaceae family. Approximately 80 species of *L. barbarum* L exist globally, with a predominant concentration on the American continent (Yao et al., 2021). In China, *L. barbarum* L. has a recorded history exceeding 3,700 years and a cultivation history of >600 years (Yu et al., 2023). *Lycium Chinense Mill* is a representative variety among them, known for its excellent quality (Yang et al., 2022). *Lycium barbarum* L., a nutrient-rich edible medicinal plant, is essential in TCM and has a history of >2000 years as a TCM and food supplement (Yun et al., 2022). It possesses properties that nourish the liver and kidneys, replenish essence, and improve eyesight (Ou et al., 2025). Modern scientific research has clarified the potential of *L. barbarum* L. in protecting the retina and impeding cell apoptosis (Liu et al., 2022). The active metabolites of *L. barbarum* L. can effectively mitigate retinal damage and improve visual function through various mechanisms, including antioxidant, anti-inflammatory, anti-apoptotic properties, and the promotion of nerve regeneration (Jiang et al., 2024). *Lycium barbarum* L. is rich in various bioactive metabolites, including *L. barbarum* polysaccharides (LBP), carotenoids, flavonoids, vitamins (including vitamins A, C, and E), and mineral elements, collectively contributing to its unique nutritional and medicinal value system (Lee and Choi, 2023). Among these metabolites, LBP, carotenoids, and flavonoids are not only abundant, but also play a significant role in the medicinal value of *L. barbarum* L.

Although the potential of *L. barbarum* L. in protecting retinal cells has been confirmed, investigations into its specific mechanism of action are still relatively scarce. Therefore, this study aims to systematically clarify the pharmacological mechanisms of *L. barbarum* L. and its active metabolites, including LBP, carotenoids, and flavonoids, in preventing and treating retinal cell apoptosis, thereby providing a comprehensive theoretical basis for enhancing their medicinal value in ophthalmology.

## 2 Methodology

In order to explore the potential efficacy and mechanism of *L. barbarum* L. as a botanical drug for preventing and treating retinal cell apoptosis, we mainly searched relevant articles in databases such as PubMed, Web of Science, and Google Scholar. The search strategy included nine keywords: “*L. barbarum* L.”, “*Lycium Chinense Mill*”, “goji berry”, “Gouqizi”, “*L. barbarum* polysaccharides”, “carotenoids”, “flavonoids”, “traditional Chinese medicine”, and “mechanism”. Two independent reviewers evaluated each article based on predefined inclusion and exclusion criteria. The literature search period was from January 2010 to January 2025. The inclusion criteria were: (1) Articles written in English; (2) Articles published in peer-reviewed journals; (3) Articles that studied the relevant mechanisms and clinical applications of *L. barbarum* L. and its active metabolites in preventing and treating retinal cell apoptosis. The exclusion criteria were: (1) Non-English articles; (2) Non-peer-reviewed articles, such as conference abstracts, editorials, and non-academic reports; (3) Articles that had nothing to do with *L. barbarum* L. and its active metabolites; (4) Duplicate articles.

## 3 The main active metabolites of *Lycium barbarum* L.

### 3.1 LBP

LBP, as a distinctive bioactive metabolite in *L. barbarum* L., has a complex chemical structure comprising various monosaccharides, including mannose, xylose, galacturonic acid, glucose, galactose, and arabinose (Yin et al., 2024). These sugar chain structures endow LBP with unique biological activities, including immune regulation, antioxidant properties, hypoglycemic effects, and lipid-lowering capabilities, which are essential to the pharmacological effects of *L. barbarum* L. (Liu et al., 2024b; Wu D. et al., 2024).

**FIGURE 1:**
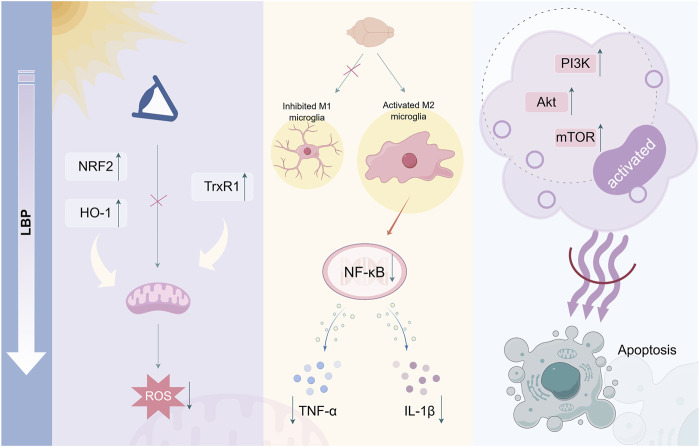
Mechanism of LBP in inhibiting retinal cell apoptosis. LBP inhibits ROS generation and reduces cellular oxidative damage by upregulating antioxidant genes. Simultaneously, it can inhibit the expression of pro-inflammatory cytokines TNF-α and IL-1β through NF-κB signaling. Additionally, LBP can activate the anti-apoptotic signaling pathway PI3K/Akt/mTOR, exerting anti-apoptotic effects By Figdraw.

Regarding retinal protection, LBP exhibits significant inhibitory effects on retinal cell apoptosis through various mechanisms (Figure 1). A previous study has demonstrated that LBP can effectively prevent ROS generation in the retina of mice exposed to light, potentially through the upregulation of antioxidant genes NRF2 and TrxR1, attenuating mitochondrial responses to oxidative stress and enhancing antioxidant capacity (Tang et al., 2018). This regulatory effect weakens the mitochondrial response to oxidative stress, thereby enhancing antioxidant capacity and offering effective protection for photoreceptor cells against light-induced retinal damage. Furthermore, LBP may protect ARPE-19 cells from oxidative stress damage caused by hydrogen peroxide by activating the NRF2/heme oxygenase 1 (HO-1) signaling pathway, demonstrating significant efficacy in reducing oxidative damage and inhibiting cell apoptosis (Liang et al., 2021). The strong antioxidant capacity of LBP can eliminate excessive ROS and free radicals generated in retinal cells, thereby mitigating oxidative stress damage and protecting retinal cells from apoptosis.

LBP directly mitigates oxidative stress and indirectly protects the retina by regulating immune cell function. Microglia in the retina are immune cells of the central nervous system that typically oversee and maintain tissue homeostasis (Li L. et al., 2021). Once overactivated, they release a significant amount of inflammatory mediators, resulting in retinal inflammation and damage (Zhang et al., 2022). LBP can regulate microglial activities, inhibit their polarization toward the pro-inflammatory M1 phenotype, facilitate their transformation toward the anti-inflammatory M2 phenotype, and consequently enhance the survival rate of retinal ganglion cells (Li et al., 2019). LBP diminishes neuroinflammation and mitigates chronic retinal inflammation to protect visual function by inhibiting microglial phagocytosis and migration, decreasing the release of inflammatory mediators, including TNF-α and IL-1β, and inhibiting NF-κB signaling pathway activation (Ni et al., 2024). Furthermore, LBP can activate anti-apoptotic signaling pathways in retinal cells, including phosphatidylinositol 3-kinase (PI3K)/protein kinase (Akt)/mammalian target of rapamycin (mTOR), enhance cell survival factor expression, and inhibit the activation of apoptosis-related proteins, directly combating the process of cell apoptosis (Qi et al., 2022). LBP has potential therapeutic value in retinal degenerative diseases, including RP and AMD, which are characterized by retinal cell apoptosis.

### 3.2 Carotenoids

*Lycium barbarum* L. is abundant in carotenoids, including β-carotene, lutein, and zeaxanthin. (Neelam et al., 2021), which are complex chemical structures composed of various carbon-hydrogen chains and functional groups, including hydroxyl and ketone groups (Li L. H. et al., 2020).

β-Carotene is crucial for the conversion to retinol in the body, which is essential for retinal photoreceptor cells (Zhong et al., 2023). Retinol, the active form of vitamin A, binds with opsin to form rhodopsin, an essential compound for retinal rod cells to detect low light (Fujiki et al., 2022). Upon exposure to light, rhodopsin decomposes into opsin and retinol, initiating the generation of neural signals and facilitating vision (Radhakrishnan et al., 2024). In the darkness, vitamin A can re-synthesize rhodopsin, maintaining the sensitivity of the retina to dim light. Therefore, the intake of β-carotene is essential for the synthesis of rhodopsin, ensuring the normal function of rod cells, homeostasis of retinal retinas, and promoting visual health. Recent studies have demonstrated that β-carotene can significantly mitigate the pathological structural damage of retinal tissue during RP disease, inhibit the secretion of inflammatory factors including NF-κB, TNF-α, interleukin-6 (IL-6), and IL-1β (Shi et al., 2024), reduce oxidative metabolite accumulation (Li et al., 2017), and effectively restore cone cell function (Moon et al., 2023).

The retina is exposed to light for a long time, particularly blue light, which has high energy and can directly penetrate the eyeball to reach the retina. Excessive blue light can induce photooxidative stress and damage retinal cells (Ziółkowska and Lewczuk, 2022). Lutein and zeaxanthin are densely concentrated in the macular region of the retina and can absorb light within a specific wavelength range (Masri et al., 2024). This optical property enables the filtration of harmful light, including blue light, and reduces photooxidative damage to the retina (Jia et al., 2017). From a biological perspective, the mechanism through which lutein and zeaxanthin protect retinal cells from apoptosis is associated with the regulation of antioxidant capacity and signaling pathways. They engage in the intracellular antioxidant defense system, capturing and neutralizing free radicals through their structure, protecting the lipids, proteins, and nucleic acids of retinal cells from oxidative stress damage (Rozanowska et al., 2023). Additionally, they can regulate cell signaling and maintain the normal order of metabolism, proliferation, and differentiation of retinal cells, thereby protecting the structural and functional integrity of cells and reducing the risk of apoptosis (Liu et al., 2021).

**FIGURE 2:**
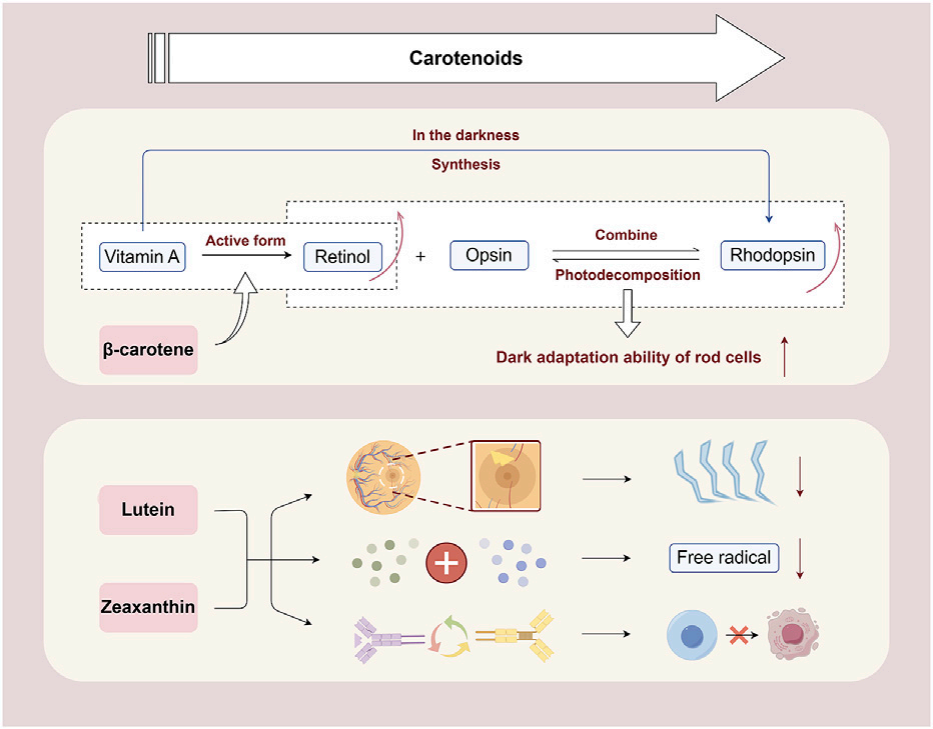
Mechanism of carotenoids in inhibiting retinal cell apoptosis. β-Carotene can help promote the conversion of retinol in the body, maintain the synthesis of rhodopsin, ensure the normal function of rod cells, and promote visual health. Lutein and zeaxanthin can filter blue light and reduce photooxidative damage to the retina. They neutralize free radicals through their own structure and protect retinal cells from oxidative stress damage. Besides, they can regulate cell signaling, maintain normal metabolism, proliferation, and differentiation of retinal cells, and reduce the risk of apoptosis By Figdraw.

Because of their essential role in protecting retinal photoreceptor cells from apoptosis (Figure 2), carotenoids have exhibited significant potential in various retinal disease treatments and prevention. In retinal degenerative diseases characterized by photoreceptor cell apoptosis, including RP, β-carotene can effectively preserve patients’ dark adaptation ability, expand the visual field, and correct vision (Rotenstreich et al., 2013). Zeaxanthin dipalmitate can markedly improve the survival rate of photoreceptors, improve retinal photo response, and mitigate morphological and functional degeneration of the retina (Chen et al., 2024). In AMD, supplementation with carotenoids (especially lutein and zeaxanthin) slows down the geographic atrophy progression of AMD toward the fovea (Keenan et al., 2025) while protecting visual function (Li X. et al., 2021).

### 3.3 Flavonoids

Flavonoids are a class of essential secondary metabolites widely distributed and abundant in foodborne plants (Liu et al., 2023). Previous studies demonstrated that the flavonoids in *L. barbarum* L. are primarily composed of rutin, quercetin, kaempferol, isorhamnetin, and luteolin, which exhibit various pharmacological effects, including antioxidant, anti-inflammatory, and vasodilatory properties (Mocan et al., 2019; Li T. et al., 2020).

**FIGURE 3:**
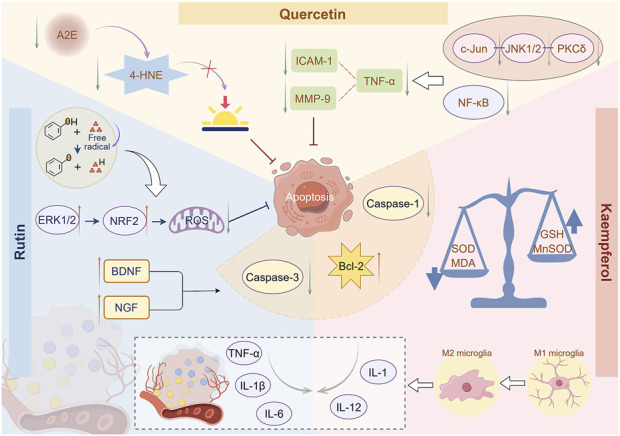
Mechanism of flavonoids in inhibiting retinal cell apoptosis. Rutin can enhance NRF2 expression by activating the ERK1/2 signaling pathway, thereby inhibiting the production of ROS in retinal cells. Rutin can reduce the levels of inflammatory factors, including TNF-α, IL-1β, and IL-6, in tissues, thereby alleviating inflammatory stimulation on retinal cells. Rutin can promote the expression of BDNF and NGF, thereby exhibiting anti-apoptotic activity. Quercetin inhibits A2E formation, reduces 4-HNE release, and thus prevents photooxidation processes in the retina. Quercetin can inhibit inflammatory signaling pathways and reduce the levels of TNF-α, ICAM-1, and MMP-9, thereby suppressing inflammatory responses. Kaempferol can significantly reduce the levels of ROS and MDA and increase the levels of GSH and MnSOD, thereby reducing cellular oxidative stress damage. Kaempferol can reduce the production of inflammatory factors TNF-α and IL-1β and significantly lower the levels of Bcl-2, caspase-1, and caspase-3, thereby inhibiting retinal cell apoptosis By Figdraw.

Rutin has excellent antioxidant properties because the phenolic hydroxyl groups in its molecular structure can provide hydrogen atoms to bind with free radicals, rendering them inactive, reducing oxidative stress damage to cells, and protecting human cells and tissues (Safdari et al., 2022). A previous study has demonstrated that rutin can enhance NRF2 expression by activating the extracellular signal-regulated protein kinases one and 2 (ERK1/2) signaling pathway in RPE cells (Li Y. et al., 2021). It improves tert-butyl hydroperoxide-induced cell death and promotes cell viability by inhibiting the generation of intracellular ROS, demonstrating its potential value in preventing retinal diseases caused by oxidative damage. Some studies have demonstrated that rutin treatment significantly reduces the tortuosity index observed during fundus examination (Gupta et al., 2020), enhances the diameter of retinal arterioles, and reduces the concentrations of pro-inflammatory cytokines, including TNF-α, IL-1β, and IL-6 in tissues (Moldovan et al., 2023). This indicates that rutin may enhance the toughness of retinal capillaries, diminish their permeability, and alleviate inflammatory stimuli on endothelial cells while preserving the integrity of the vascular wall (Palmitessa et al., 2022). Additionally, rutin can inhibit cell apoptosis by modulating intracellular signaling pathways (Ma et al., 2017). It can enhance the expression of cell survival factors, including brain-derived neurotrophic factor (BDNF) and nerve growth factor (NGF), diminish the level of caspase-3 in the retina, and increase the level of B-cell lymphoma-2 (Bcl-2), thereby demonstrating anti-apoptotic activity (Ola et al., 2015). This mechanism of action renders flavonoids significant in delaying the degeneration of retinal photoreceptor cells (Figure 3).

With advancing age, the dual retinal fluorescent compound A2E, a photoresponsive retinal aldehyde derivative, gradually accumulates in RFE cells (Arunkumar and Bernstein, 2023). Quercetin has been shown to inhibit the formation of photooxidative A2E species from the source, reduce the release of lipid peroxidation product 4-hydroxynonenal (4-HNE), and consequently prevent the photooxidative process in the retina (Wang et al., 2017). Quercetin exhibits anti-inflammatory properties by inhibiting inflammatory signaling pathways, including PKCδ-JNK1/2-c-Jun and NF-κB, decreasing the synthesis of inflammatory mediators, including TNF-α, and suppressing the expression and activity of intercellular adhesion molecule-1 (ICAM-1) and matrix metalloproteinase-9 (MMP-9), thus mitigating the recruitment and dissemination of inflammatory cells (Cheng S. et al., 2019).

The chemical structural similarity between kaempferol and quercetin endows them with similar biological characteristics and functions. It can act on immune cells, regulate cytokine secretion, maintain an appropriate level of the immune response, and prevent excessive immune response leading to inflammation (Tanaka et al., 2022). Diabetic retinopathy (DR) is a common secondary complication of diabetes. The immune system is involved in DR, which affects microglia-mediated retinal immune response (Lim et al., 2024). In the initial phase, it is characterized by the permeability of the blood-retinal barrier, facilitating interaction between the peripheral and retinal immune systems (Kim et al., 2024). A previous study demonstrated that kaempferol treatment promotes phenotypic and functional changes in immune cells, strongly inhibits pro-inflammatory responses during DR progression, diminishes the synthesis of inflammatory factors TNF-α and IL-1β, and significantly reduces the levels of Bcl-2, caspase-1, and caspase-3, consequently inhibiting retinal cell apoptosis (Albalawi et al., 2024). In RPE cells, kaempferol has been shown to significantly reduce the levels of ROS and lipid peroxidation product malondialdehyde (MDA), while increasing the levels of endogenous antioxidants glutathione (GSH) and manganese superoxide dismutase (MnSOD) (Al Sabaani, 2020). Kaempferol can also reduce the production of inflammatory factors including interleukin-1 (IL-1), IL-6, and interleukin-12 (IL-12), enhance RPE cell proliferation activity, and thereby reduce RPE cell apoptosis (Akalın and Selamoglu, 2019; Zhang et al., 2024). However, kaempferol can effectively modulate endothelial cell function, stimulate nitric oxide release, induce vasodilation, improve local blood perfusion, and deliver sufficient nutrients to tissues and organs (Firoz and Talwar, 2022). Experimental results revealed that in a mouse model of oxygen-induced retinopathy, kaempferol inhibited retinal neovascularization and effectively prevented VEGF-induced excessive retinal vascular permeability (Jung et al., 2020). Other flavonoids, including isorhamnetin, have demonstrated similar protective effects that may delay retinal disease progression (Hui et al., 2024).

## 4 The main mechanisms of retinal cell apoptosis and the targets of *Lycium barbarum* L. action

### 4.1 Oxidative stress mechanism and antioxidant intervention of *Lycium barbarum* L.

During evolution, cells have developed a complete antioxidant defense system. The system includes antioxidant enzymes, such as superoxide dismutase (SOD) and glutathione peroxidase (Selamoglu et al., 2017), which specifically catalyze the dismutation, decomposition, and other reactions of ROS, thereby reducing their concentration (Wang Y. et al., 2021). Concurrently, there are non-enzymatic antioxidant enzymes, including GSH, vitamin C, and vitamin E, which utilize their intrinsic chemical reduction properties to directly neutralize ROS (Monsalves et al., 2020). Under normal physiological conditions, the two work collaboratively to regulate ROS, maintain cellular redox homeostasis, and prevent oxidative damage (Georgescu et al., 2022). However, in a pathological state, the function of the antioxidant defense system is impaired, leading to an imbalance, ROS accumulates in large quantities, and cell damage is exacerbated (Zhou et al., 2021).

The retina is the fundamental region of visual perception, where photoreceptor cells play a crucial role in the initial stage of visual signal transduction (Takeda et al., 2022). Photoreceptor cells exhibit a unique physiological characteristic of a high metabolic rate aimed at fulfilling the energy demands required for visual signal capture and conversion (Oltra et al., 2022). Simultaneously, their microenvironment exhibits a high oxygen partial pressure. This unique metabolic and environmental condition renders photoreceptor cells highly susceptible to oxidative stress (Santhanam et al., 2023). The complex pathological mechanism of retinal lesions involves a disruption of internal homeostasis, leading to a substantial increase in ROS and free radicals (Yang et al., 2020; Al-Sroji et al., 2023), including superoxide anions and hydroxyl radicals (Selamoglu et al., 2020), which possess potent oxidative properties and specifically target photoreceptor cells (Garza et al., 2024). Initially, ROS trigger lipid peroxidation reactions, compromising the lipid bilayer architecture of the cell membrane, resulting in impaired membrane integrity, decreased fluidity, and severe disruption of substance exchange and signal transmission intracellularly and extracellularly (Sui et al., 2024). At the molecular level, ROS can modify amino acid residues, causing conformational changes in proteins and resulting in the loss of their original physiological functions, thereby impeding numerous protein-dependent metabolic pathways within cells. Oxidative damage can adversely affect DNA, leading to base pair mismatches, deletions, and DNA strand breaks, resulting in errors in the storage and transmission of genetic information (Kumar et al., 2022). These oxidative stress damage events are interconnected and progressively activate the meticulously regulated apoptotic signaling pathway within cells, creating latent risk for photoreceptor apoptosis and endangering visual function (Huang et al., 2024).

**FIGURE 4:**
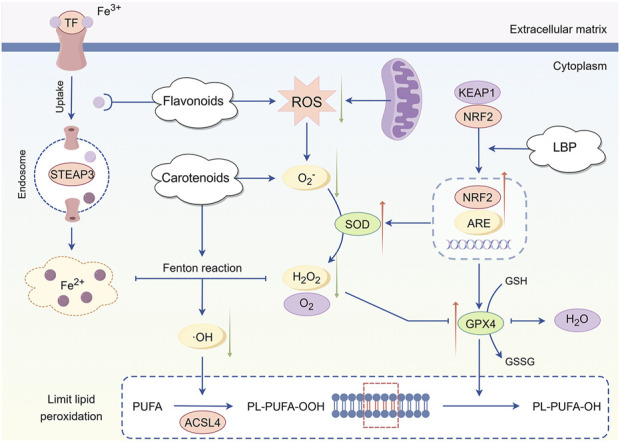
Mechanism of antioxidant intervention in *Lycium barbarum* L. LBP facilitates the dissociation of NRF2 from KEAP1, leading to the activation of NRF2 and its translocation into the nucleus. This process initiates the transcription and expression of antioxidant enzyme genes, including SOD and GPX4. SOD catalyzes the dismutation of superoxide anions into hydrogen peroxide and oxygen, while GPX4 further reduces hydrogen peroxide to water. Together, these enzymes effectively mitigate excess ROS within cells, thereby reducing oxidative stress at its source and maintaining the redox homeostasis of the photoreceptor cell environment. Additionally, carotenoids directly neutralize free radicals, converting them into stable products, which diminishes the direct assault of free radicals on photoreceptor cells. Flavonoids contribute by chelating metal ions, thereby inhibiting ROS production By Figdraw.

The various active metabolites in *L. barbarum* L. exhibit antioxidant properties (Figure 4). NRF2 is the principal transcription factor governing the cellular antioxidant stress response (Jin et al., 2024). Under normal conditions, NRF2 binds to kelch-like ECH-associated protein 1 (KEAP1) and remains in an inactive state within the cytoplasm (Ulasov et al., 2022). Under oxidative stress, photoreceptor cells utilize LBP to specifically bind and modify KEAP1, facilitating the uncoupling of NRF2 and KEAP1, which subsequently activates and translocates NRF2 into the nucleus (Hu et al., 2021; Nguyen et al., 2024). NRF2 translocates to the nucleus, precisely binds to antioxidant response elements (ARE) (Choublier et al., 2022) and activates a cascade of transcription programs for antioxidant enzyme genes, including SOD and glutathione peroxidase 4 (GPX4), leading to their substantial expression (Yu and Xiao, 2021). SOD catalyzes the dismutation of superoxide anions into hydrogen peroxide and oxygen, while GPX4 subsequently reduces hydrogen peroxide to water. The two collaborate to efficiently eliminate excess ROS in cells, reducing oxidative stress at its origin and maintaining the redox homeostasis of the photoreceptor cell environment (García-Pérez et al., 2021). Carotenoids can directly neutralize free radicals, and their polyene chain structure can interact with free radicals to transform them into stable products, thereby reducing the direct assault of free radicals on photoreceptor cells (Welc-Stanowska et al., 2023). Flavonoids can inhibit ROS generation reactions catalyzed by metal ions, including iron and copper ions, by chelating them (Vučkovski et al., 2024). Simultaneously, they can enhance the activity of other antioxidant components, synergistically producing antioxidant effects and inhibiting the oxidative stress-induced apoptosis process of photoreceptor cells through multiple pathways (Shao et al., 2019).

### 4.2 Inflammatory response mechanism and anti-inflammatory regulation of *Lycium barbarum* L.

**FIGURE 5:**
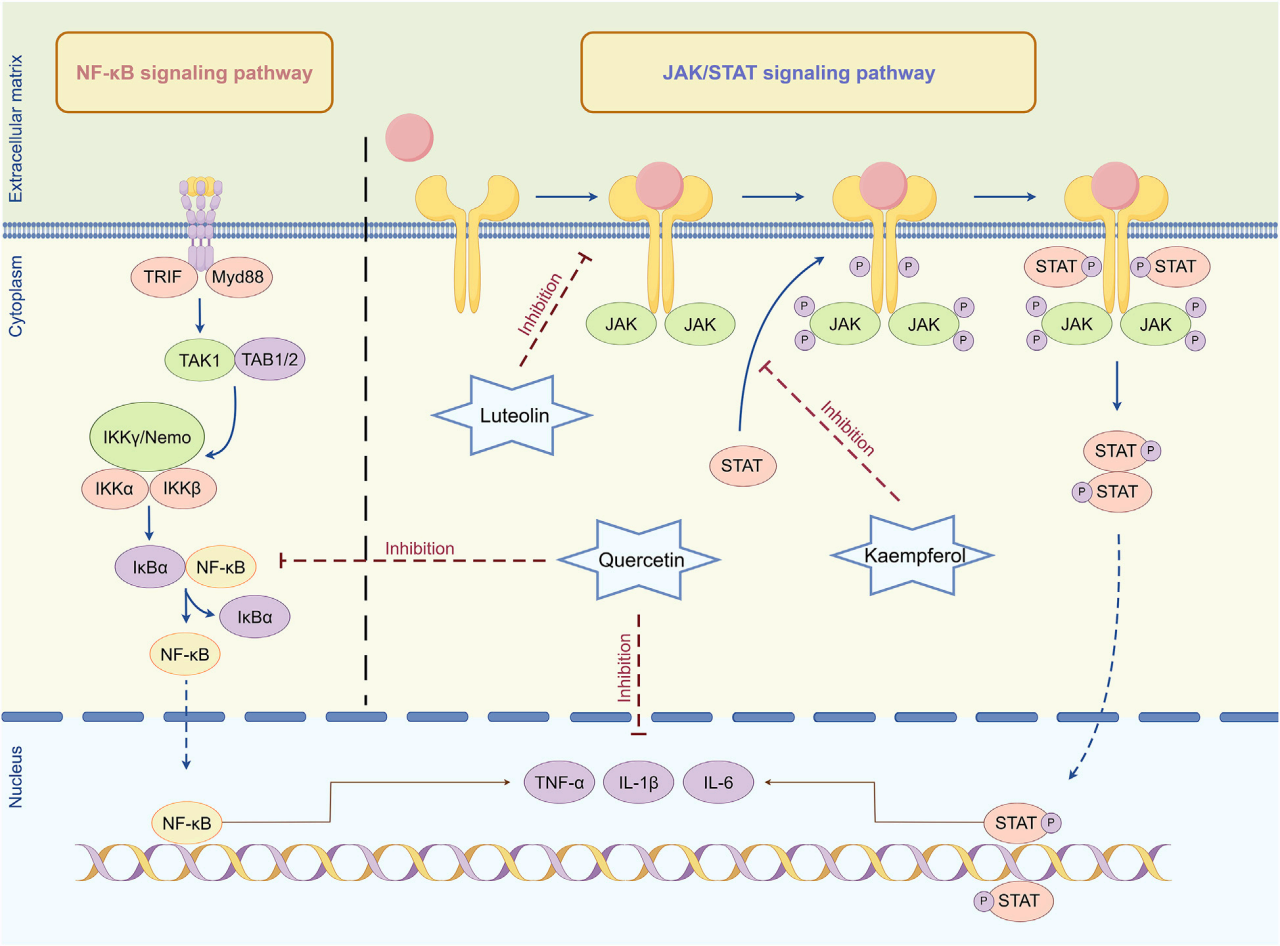
Mechanism of anti-inflammatory regulation in *Lycium barbarum* L. Quercetin can inhibit the activation of NF-κB and JAK/STAT signaling pathways, reduce the production and release of inflammatory factors, and thus alleviate the damage of inflammatory response to retinal cells. Luteolin can interfere with the binding of cytokines to their corresponding receptors, thereby preventing receptor dimerization and JAK recruitment, thereby inhibiting JAK activation. Kaempferol can inhibit the phosphorylation of receptor tyrosine by activating JAK, blocking the formation of STAT docking sites and inhibiting signal transduction By Figdraw.

When the retina is damaged or infected, immune cells, including microglia and macrophages, become activated, secreting a substantial amount of inflammatory factors, including TNF-α, IL-1β, and IL-6 (Wang et al., 2025). These inflammatory factors further elicit inflammatory responses in retinal cells by activating downstream signaling pathways, including NF-κB and janus kinase (JAK)/signal transducer and activator of transcription (STAT), resulting in cellular damage and apoptosis (Figure 5).

NF-κB is a nuclear transcription factor prevalent in eukaryotic cells, crucial for regulating multiple biological processes, including immune response, inflammatory response, cell proliferation, differentiation, and apoptosis (Ravichandran and Selamoglu, 2023; Cornice et al., 2024). NF-κB typically exists in an inactive state within cells and binds to its inhibitory protein inhibitor of NF-κB (IκB) in the cytoplasm (Du et al., 2024). When cells encounter diverse stress stimuli, including ultraviolet radiation, oxidative stress, or cytokines, these stimuli activate signal transduction pathways, resulting in the phosphorylation of IκB and subsequent proteasomal degradation (Mamun et al., 2024). This process facilitates NF-κB release and translocation into the nucleus, where it binds to the promoter regions of specific genes, thereby enhancing their transcription and expression (Shahbazi and Zakerali, 2022). Activation of NF-κB in retinal cells enhances the transcription and expression of various inflammatory genes. These genes encode cytokines, chemokines, adhesion molecules, and acute phase response proteins (Ando et al., 2020). The active metabolites in *L. barbarum* L., including quercetin, can inhibit the activation of the NF-κB signaling pathway, diminish the synthesis and release of inflammatory factors, and thereby mitigate the detrimental effects of the inflammatory response on retinal cells (Cheng S. C. et al., 2019).

Additionally, the JAK/STAT signaling pathway is essential in the inflammatory response of retinal cells alongside the NF-κB signaling pathway. The JAK/STAT signaling pathway is a series of reactions involving interactions among intracellular proteins, primarily involved in processes including immunity, cell division, apoptosis, and tumorigenesis (Xue et al., 2023). Activation of the JAK/STAT signaling pathway enhances the transcription and expression of inflammatory factors, thereby exacerbating the inflammatory response and resulting in damage and dysfunction of retinal cells (Chen et al., 2022). A previous study has demonstrated that the active metabolites in *L. barbarum* L. can inhibit the activation of the JAK/STAT signaling pathway. Luteolin can obstruct the binding of cytokines to their respective receptors, thus hindering receptor dimerization and JAK recruitment, thereby inhibiting JAK activation (Tai et al., 2014). Subsequently, kaempferol can inhibit the phosphorylation of receptor tyrosine by activating JAK, thereby obstructing the formation of STAT docking sites and further inhibiting STAT activation and subsequent signal transduction (Li et al., 2023). By inhibiting the activation of the JAK/STAT signaling pathway, quercetin diminishes the transcription and translation of inflammatory factors, thereby mitigating the detrimental effects of the inflammatory response on retinal cells, which aids in preventing and treating inflammation-related retinal diseases (Zou et al., 2024).

### 4.3 Abnormal autophagy mechanism and regulatory intervention of *Lycium barbarum* L.

Autophagy is a cellular self-degradation process involving autophagosome formation with a bilayer membrane structure, which encapsulates and degrades proteins, organelles, and other substances to maintain homeostasis (Dragowska et al., 2024). This process is essential in various physiological and pathological mechanisms, including cell survival, development, immunity, and disease manifestation (Zapatería and Arias, 2024). However, the dysregulation of autophagy may lead to impaired retinal cell function, potentially precipitating various retinal diseases (Wang X. et al., 2024).

**FIGURE 6:**
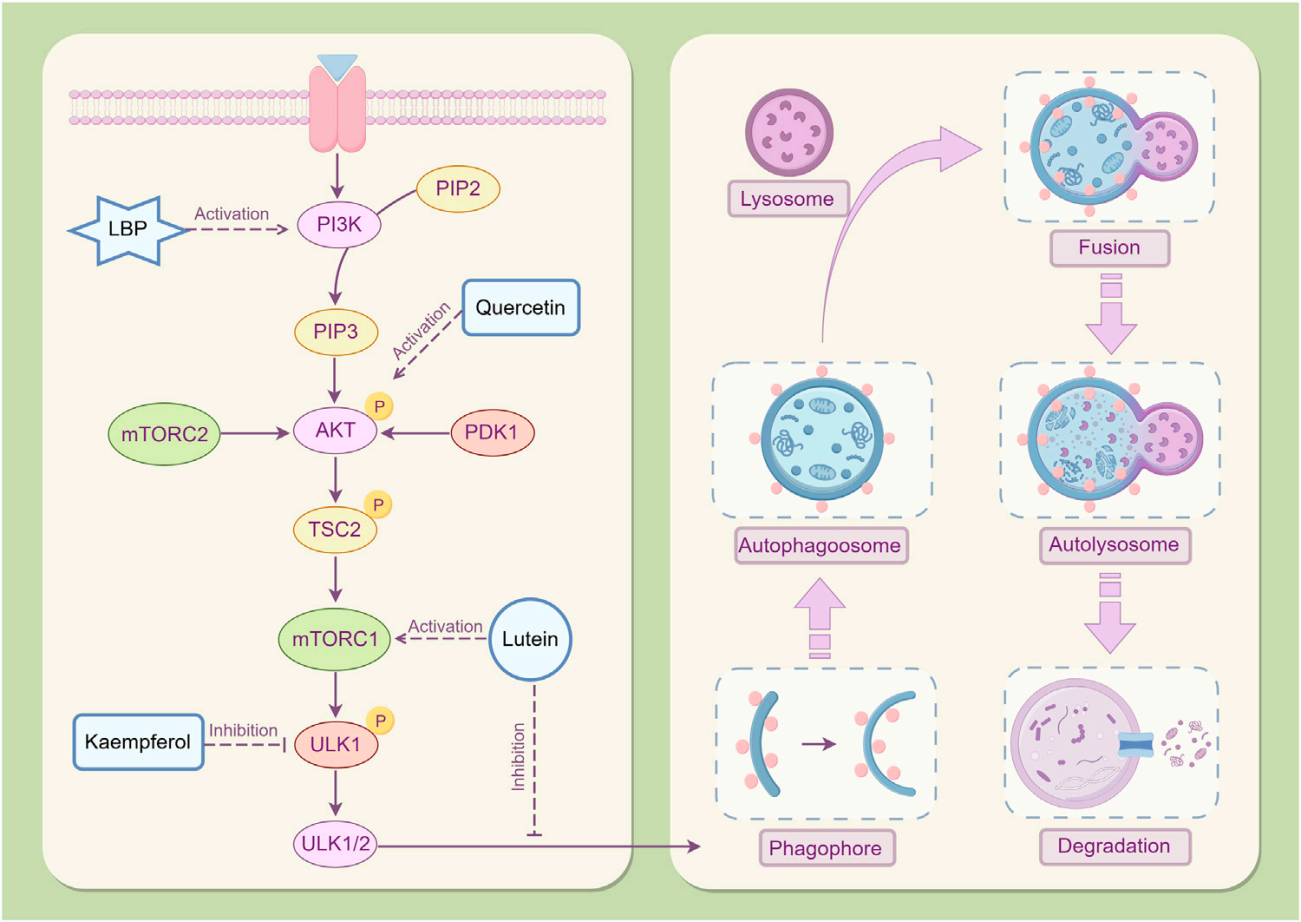
Mechanism of autophagy regulation in *Lycium barbarum* L. LBP mitigates cellular damage induced by excessive autophagy through the activation of the PI3K/Akt/mTOR signaling pathway, modulation of autophagy-related gene expression, and facilitation of autophagosome formation. Lutein exerts its effects by inhibiting autophagosome formation via mTOR activation, thereby reducing cellular apoptosis. Quercetin has been demonstrated to significantly elevate phosphorylated Akt protein levels in the retina. Moreover, Kaempferol has exhibited efficacy in inhibiting the phosphorylation of mTOR and ULK1, thereby facilitating the removal of senescent and damaged cellular components By Figdraw.

The PI3K/Akt/mTOR signaling pathway is essential for anti-apoptotic processes. It is a primary regulator of cellular autophagy (Figure 6), which is regulated by sensing the nutritional and energy status inside the cell (Demir et al., 2024). PI3K is the starting point for signal transduction, catalyzing phosphatidylinositol 4,5-bisphosphate (PIP2) to generate phosphatidylinositol 3,4,5-trisphosphate (PIP3), which functions as a second messenger to activate downstream signaling molecules (Li et al., 2022). Akt, a direct downstream target of PI3K, is activated by the recruitment of PIP3 and further activates mTOR through phosphorylation, initiating a cascade reaction (Sun et al., 2022). In a nutrient-rich environment, the high energy and abundant nutrients within cells activate the PI3K/Akt/mTOR signaling pathway (Liu and Sabatini, 2020). As the terminal effector molecule of this pathway, the activated state of mTOR effectively inhibits autophagy by inhibiting the activity of the unc-51-like kinase 1/2 (ULK1/2) complex (autophagy initiating complex) and hindering the formation of autophagosomes (Despotović et al., 2022). This process guarantees that cells can completely exploit the nutritional resources available from their environment to facilitate their growth and proliferation requirements. However, under conditions of nutrient deficiency or injury stress, the activity of the PI3K/Akt signaling pathway weakens, resulting in the dephosphorylation and subsequent inactivation of mTOR (Zi et al., 2022). The mTOR-mediated inhibition of autophagy is subsequently alleviated, facilitating the onset of autophagy (Wang and Zhang, 2019). The ULK1/2 complex is activated, initiating the expression of autophagy-related genes and facilitating the formation and maturation of autophagosomes (Liang et al., 2023). Excessive autophagy can result in the excessive degradation of important nutrients and proteins within cells, resulting in ineffective clearance of autophagosomes, which can compromise cellular structure and function, potentially initiating apoptotic pathways (Wang L. et al., 2021).

The PI3K/AKT/mTOR signaling pathway is highly activated in RPE cells during proliferative vitreoretinopathy (Cai et al., 2012). LBP can enhance the survival and proliferation of retinal cells by activating the PI3K/Akt/mTOR signaling pathway (Qi et al., 2022) and can inhibit excessive autophagy in RPE cells, thereby safeguarding them from light-induced damage (Gao et al., 2022). This activating effect preserves the nutritional and energy balance within cells, thus preventing cellular damage from excessive autophagy by regulating the expression of autophagy-related genes and the formation of autophagosomes (Yu et al., 2018). Additionally, lutein inhibits the formation of autophagosomes after hypoxia injury by activating the mTOR signaling pathway, thereby enhancing the survival rate of Müller cells and inhibiting cell apoptosis (Fung et al., 2016). After quercetin intervention, the phosphorylated Akt protein level in the diabetic retina increased significantly (Ola et al., 2017). Quercetin may protect neurons in the diabetic retina from damage by inhibiting neuronal apoptosis. Kaempferol was reported to exhibit good effects in mTOR inhibition and ULK1 phosphorylation to eliminate aging and damaged components (Salehi et al., 2018; Kim et al., 2023).

### 4.4 Mitochondrial dysfunction and the protective effect of *Lycium barbarum* L.

Mitochondria are the cellular organelles that generate adenosine triphosphate (ATP) and maintain normal physiological functions within cells (Botchway et al., 2022). However, in numerous disease states, the mitochondrial function can be compromised, leading to insufficient energy supply, increased oxidative stress, and apoptosis, a phenomenon known as mitochondrial dysfunction (Huang et al., 2023). For retinal cells, a lack of sufficient energy impedes their ability to maintain normal physiological functions, leading to a significant decrease in the efficiency of capturing and converting light signals and a deceleration or interruption in the transmission of nerve signals (Zhang et al., 2023). This series of chain reactions will ultimately exert a severe impact on the visual system. Mitochondria are energy producers and the main source of intracellular ROS. The impaired mitochondrial function may lead to an abnormal increase in ROS production while the cellular capacity to eliminate ROS diminishes, significantly increasing intracellular oxidative stress levels (Zhao et al., 2022). Oxidative stress is a deleterious process that further damages the DNA, proteins, and membrane structure of mitochondria, forming a vicious cycle where mitochondrial dysfunction increases ROS, thereby exacerbating mitochondrial damage (Sule et al., 2022).

Additionally, mitochondria regulate intracellular calcium ions, which is crucial for maintaining cellular homeostasis (Guo et al., 2023). Impaired mitochondrial function can severely disrupt calcium ion homeostasis, resulting in an abnormal increase in intracellular calcium ion concentration, which may trigger apoptosis or necrosis of retinal cells (Yan et al., 2022). This is an irreversible process of cell demise, whether through programmed apoptosis or necrosis caused by physical or chemical injury, which can lead to a significant decline or complete loss of retinal function.

**FIGURE 7:**
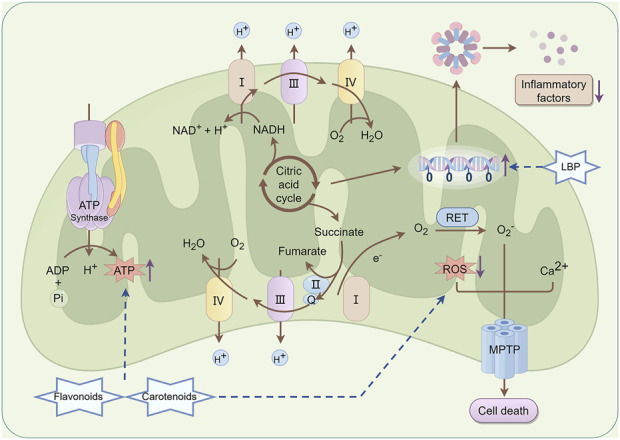
Mechanism of mitochondrial protection in *Lycium barbarum* L. LBP can effectively mitigate mitochondrial DNA damage, enhance membrane potential, and restore respiratory chain function, thereby improving the energy supply and physiological function of photoreceptor cells. Besides, LBP can inhibit the inflammatory response induced by photodamage and reduce further cellular damage from inflammatory factors. Carotenoids and flavonoids contribute to promoting ATP synthesis and provide adequate energy support for photoreceptor cells. Furthermore, they reduce ROS production within mitochondria, thereby preserving the structural and functional integrity of mitochondria and interrupting the critical pathway of retinal cell apoptosis at the mitochondrial level By Figdraw.

A previous study demonstrated that LBP significantly ameliorates mitochondrial dysfunction in retinal cells induced by light damage (Wu F. et al., 2024). In the light damage model, LBP can significantly mitigate mitochondrial DNA damage, enhance membrane potential, and restore respiratory chain function, thereby enhancing the energy supply and physiological function of photoreceptor cells. In addition, LBP can inhibit the inflammatory response induced by light damage and mitigate subsequent cellular damage caused by inflammatory factors (Ni et al., 2024). Carotenoids and flavonoids, the main active metabolites in *L. barbarum* L., may optimize the energy metabolism pathway of mitochondria, promote ATP synthesis, and provide sufficient energy for photoreceptor cells (Pan et al., 2019; Bu et al., 2020). These metabolites can diminish the generation of ROS in mitochondria, safeguard the structural and functional integrity of mitochondria, and consequently inhibit the critical pathway of retinal cell apoptosis at the mitochondrial level (Imran et al., 2019; Ademowo et al., 2024). The specific mechanism is shown in Figure 7.

## 5 Conclusion and perspectives

Compared with TCM treatment methods, modern medicine for retinal diseases often targets a single point. When faced with the complex physiological and pathological processes of the retina, single-target treatment is difficult to address all aspects of the disease comprehensively. In contrast, multiple metabolites in *L. barbarum* L. can work together to treat retinal diseases. LBP has a unique immune-regulating function, which not only enhances the self-repair ability of retinal cells but also plays a vital role in autophagy regulation. Carotenoids, with their strong antioxidant capacity, can quickly eliminate free radicals within retinal cells, reducing oxidative stress damage and thereby decreasing the risk of cell apoptosis. Flavonoids can suppress the release of inflammatory factors, alleviate the attack of inflammation on retinal cells, and maintain the stability of the cellular microenvironment. These metabolites intervene in retinal cell apoptosis from aspects such as immune regulation, antioxidant activity, anti-inflammation, autophagy regulation, and mitochondrial function protection and can deal with the complex disease mechanisms more comprehensively. In addition, the viewpoint of TCM holds that *L. barbarum* L. not only acts on eye cells but also regulates overall bodily functions, improves the internal environment, and prevents and treats diseases from the root cause. It effectively complements the local and targeted treatment of modern medicine and opens up new ideas for the treatment of retinal diseases.

Despite the fact that numerous scholars have verified the efficacy of *L. barbarum* L. in the treatment and prevention of retinal cell apoptosis, there are still several areas in need of improvement. Firstly, neither *in vitro* nor *in vivo* models can precisely mimic the physiological and pathological states of the retina. 2D cell cultures lack the complex immune responses and intricate intercellular interactions. Animal models suffer from species-specific differences, which pose significant challenges to accurately reflecting the real-world situation in human retinas. Secondly, there is a conspicuous absence of unified standards in the extraction and identification of *L. barbarum* L. metabolites. Different laboratories employ diverse extraction methods and experimental procedures, resulting in incomparable research outcomes. This not only hampers the cross-comparison of data but also severely obstructs the in-depth understanding of the underlying mechanisms of action of these metabolites. Thirdly, current clinical research on *L. barbarum* L. has several limitations. The number of studies is scarce, and the sample sizes are generally small. The experimental designs often lack rigor, and the follow-up periods are short. Although a series of experimental investigations have indicated that *L. barbarum* L. extracts are safe at normal doses, with no significant toxic reactions observed in animal and cell-based experiments (Mocan et al., 2018), the long-term clinical efficacy and safety of *L. barbarum* L. remain in a state of uncertainty. With the widespread use of *L. barbarum* L. in the healthcare and pharmaceutical industries, the potential toxicity at high doses, as well as the long-term cumulative effects, necessitate continuous attention. These aspects could have a profound impact on its clinical applications and safety evaluations. Fourthly, some metabolites of *L. barbarum* L. have been proven to be highly absorbable. For example, LBP can enter the bloodstream via specific transporters in the intestine, thereby exerting immune-regulating and cell-protecting functions. Nevertheless, further research is urgently required to elucidate the absorption, distribution, metabolism, and excretion of these metabolites under different individual and physiological conditions. This knowledge is crucial for optimizing the clinical utilization and therapeutic efficacy of *L. barbarum* L.

In the future, the research on *L. barbarum* L. should focus on several key directions. Developing advanced *in vitro* models such as 3D organoids or co-cultures, and conducting *in vivo* research using non-human primates that are more similar to human retinal physiology, can yield more accurate results. These approaches will enhance our understanding of the real-world effects of *L. barbarum* L. on retinal diseases. The scientific community should also collaborate to establish unified standards, making the results of different laboratories comparable and reproducible. This will promote the integration and transformation of research findings across the field. Additionally, it is of utmost importance to utilize advanced technologies such as single-cell sequencing and multi-omics to deeply explore the molecular mechanisms of *L. barbarum* L. in preventing retinal apoptosis. In terms of clinical research, large-scale, multi-center, randomized controlled trials should be carried out. By increasing the sample size, extending the follow-up time, and comprehensively evaluating the efficacy, safety, and mechanism of action of *L. barbarum* L. in preventing and treating diseases related to retinal cell apoptosis, we can gain a more comprehensive understanding of its therapeutic value. Meanwhile, exploring the combined application of *L. barbarum* L. with other botanical drugs and chemical therapies holds great promise for improving the therapeutic effect, ultimately enhancing the vision and quality of life of patients. Although *L. barbarum* L. shows great potential in the treatment of retinal diseases, continuous efforts in research methods, mechanism exploration, and clinical transformation are essential to fully realize its therapeutic value.

## Glossary

4-HNE: 4-hydroxynonenal
Akt: Protein kinase
AMD: Age-related macular degeneration
ARE: antioxidant response elements
ATP: Adenosine triphosphate
Bcl-2: B-cell lymphoma-2
BDNF: Brain-derived neurotrophic factor
DR: Diabetes retinopathy
ERK1/2: Extracellular signal-regulated protein kinases 1 and 2
GPX4: Glutathione peroxidase 4
GSH: Glutathione
HO-1: Heme oxygenase 1
ICAM-1: Intercellular adhesion molecule-1
IL-1: Interleukin-1
IL-12: Interleukin-12
IL-1β: Interleukin-1 beta
IL-6: Interleukin-6
IκB: Inhibitor of NF-κB
JAK: Janus kinase
KEAP1: Kelch-like ECH-associated protein 1
LBP: Lycium barbarum polysaccharides
MDA: Malondialdehyde
MMP-9: Matrix metalloproteinase-9
MnSOD: Manganese superoxide dismutase
mTOR: Mammalian target of rapamycin
NF-κB: Nuclear factor kappa-B
NGF: Nerve growth factor
NRF2: Nuclear factor erythroid 2-related factor 2
PI3K: Phosphatidylinositol 3-kinase
PIP2: Phosphatidylinositol 4,5-bisphosphate
PIP3: Phosphatidylinositol 3,4,5-trisphosphate
ROS: Reactive oxygen species
RP: Retinitis pigmentosa
RPE: Retinal pigment epithelial
SOD: Superoxide dismutase
STAT: Signal transducer and activator of transcription
TCM: Traditional Chinese medicine
TNF-α: Tumor necrosis factor-alpha
TrxR1: Thioredoxin reductase 1
ULK1/2: Unc-51 like kinase 1/2
